# Low Resistance to First and Second Line Anti-Tuberculosis Drugs among Treatment Naive Pulmonary Tuberculosis Patients in Southwestern Uganda

**DOI:** 10.1371/journal.pone.0118191

**Published:** 2015-02-06

**Authors:** Patrick Orikiriza, Becky Tibenderana, Mark J. Siedner, Yolanda Mueller, Frederick Byarugaba, Christopher C. Moore, Emily E. Evans, Maryline Bonnet, Anne-Laure Page, Joel Bazira, Yap Boum II

**Affiliations:** 1 Epicentre Mbarara Research Centre, Mbarara, Uganda; 2 Department of Microbiology, Mbarara University of Science and Technology, Mbarara, Uganda; 3 Department of Medicine, Mbarara University of Science and Technology, Mbarara, Uganda; 4 Department of Medicine, Massachusetts General Hospital and Harvard Medical School, Boston, United States of America; 5 Department of Medicine, Division of Infectious Diseases and International Health, University of Virginia, Charlottesville, Virginia, United States of America; 6 Epicentre Paris, Paris, France; Indian Institute of Science, INDIA

## Abstract

**Background:**

There are limited data on region-specific drug susceptibility of tuberculosis (TB) in Uganda. We performed resistance testing on specimens collected from treatment-naive patients with pulmonary TB in Southwestern Uganda for first and second line anti-TB drugs. We sought to provide data to guide regional recommendations for empiric TB therapy.

**Methods:**

Archived isolates, obtained from patients at Mbarara Regional Referral Hospital from February 2009 to February 2013, were tested for resistance to isoniazid and rifampicin using the MTBDR*plus* and Xpert MTB/RIF assays. A subset of randomly selected isolates was tested for second line agents, including fluoroquinolones (FQs), aminoglycosides, cyclic peptides, and ethambutol using the MTBDR*sl* assay. We performed confirmatory testing for FQ resistance using repeated MTBDR*sl*, the Mycobacteria growth indicator tube (MGIT) assay, and sequencing of the *gyrA* and *gyrB* genes.

**Results:**

We tested isolates from 190 patients. The cohort had a median age of 33 years (IQR 26-43), 69% (131/190) were male, and the HIV prevalence was 42% (80/190). No isolates (0/190) were rifampicin-resistant and only 1/190 (0.5%) was isoniazid-resistant. Among 92 isolates tested for second-line drug resistance, 71 (77%) had interpretable results, of which none were resistant to aminoglycosides, cyclic peptides or ethambutol. Although 7 (10%) initially tested as resistant to FQs by the MTBDR*sl* assay, they were confirmed as susceptible by repeat MTBDR*sl* testing as well as by MGIT and *gyrase* gene sequencing

**Conclusion:**

We found no MDR-TB and no resistance to ethambutol, FQs, or injectable anti-TB drugs in treatment naïve patients with pulmonary TB in Southwestern Uganda. Standard treatment guidelines for susceptible TB should be adequate for most patients with TB in this population. Where possible, molecular susceptibility testing methods should be routinely validated by culture methods.

## Background

Uganda has one of the highest rates of tuberculosis (TB) in the world, with an estimated annual incidence of 200/100,000 people [1]. National rates of multi-drug resistant TB (MDR-TB) are estimated at 1.4% among newly diagnosed and 12.1% among previously treated patients [2]. The Mbarara Regional Referral Hospital, in partnership with Ministry of Health, Uganda recently initiated an MDR-TB treatment program. However, there are limited drug resistance data from Southwestern Uganda to guide second or third line therapy regimens.

The primary objective of our study was to estimate the regional levels of TB drug resistance and to guide recommendations for treatment of naive and MDR-TB infected patients in the Southwestern region. Our *a priori* hypothesis was that there would be high rates of FQ resistance due to the widespread local use of FQs for treatment of respiratory and enteric infections [3].

## Methods

### Study site

Strains were isolated from sputum specimens collected as part of Epicentre Mbarara Research Centre diagnostic tuberculosis studies at the Mbarara Regional Referral Hospital. Participants were eligible if they had clinical suspicion of pulmonary tuberculosis, defined as ≥ 2 weeks of cough and weight loss or night sweats, during the period February 2009—February 2013. All participants were newly diagnosed TB patients at the time of sputum collection.

### Laboratory Methods

Archived isolates were thawed from -80°C storage, sub-cultured onto Lowenstein-Jensen medium and incubated at 37°C. Cultures were monitored weekly until growth was observed and then colonies were inoculated into 7H9 broth and heat killed at 95°C in a heating block for 30 minutes.

Manufacturer guidelines were followed to perform the MTBDR*plus* PCR assay for isoniazid and rifampicin resistance (Hain Lifesciences, Nehren, Germany). This assay detects mutations in the *rpoB* gene (for rifampicin resistance), the *katG* gene, and the promoter region of the *inhA* gene (for isoniazid resistance). The same isolates were also tested using the Xpert MTB/RIF assay (Cepheid Sunnyvale, USA) with a protocol modification to dilute the culture in a 1:1 ratio with saline before adding the sample (according to ITM recommendation). Additional testing of 92 randomly selected samples for second-line drug testing was performed using the MTBDR*sl* assay. This assay detects mutations in the *gyr*A gene, 16S RNA *rrs*, and *embB* genes, which confer resistance to FQ, aminoglycosides/cyclic peptides, and ethambutol, respectively.

To confirm resistance, isolates with a FQ mutation by the MTBDR*sl* assay were re-tested at the Ugandan National Tuberculosis Reference Laboratory, using Mycobacteria Growth Indicator Tube (MGIT) drug susceptibility testing for ofloxacin and kanamycin, using a threshold of susceptibility of 2ug/mL and 2.5ug/mL, respectively. Isolates with discordant results between MTBDR*sl* and MGIT were tested for the *gyr*A and *gyr*B genes of the quinolone resistance determining region (QRDR) at the Institute of Tropical Medicine in Antwerp, Belgium, where purified PCR products were sequenced with the same primers using the ABI’s Big Dye Terminator Kit (Applied Biosystems, USA) according to the manufacturer’s instructions [4]. The following mutations were considered as indicative of resistance: Thyr-80, Ala-90, Gly-88, Asp-94, Ala 90, Ala-74, Ser-91 and Ser-95, for the gyrA gene and 429bp QRDR on gyrA/B for the gyrB gene [4]. The proportion of drug resistance for each drug testing was estimated and a confidence interval of 95% was calculated using STATA version 12 (Statacorp, College Station, Texas).

### Ethical considerations

The isolates were obtained from patients enrolled in studies approved by Faculty Research and Ethics committee and the Institutional Review Board at Mbarara University of Science and Technology, and the Uganda National Council for Sciences and Technology. All patients signed informed consent to participate in the studies and any further testing on the isolates.

## Results

### Study participants

Isolates from 190 untreated study participants with positive TB sputum cultures were tested. Approximately half (93/190, 49%) were from Mbarara District and the remaining were from 22 neighboring districts in southwestern Uganda. The majority (131/190, 69%) were male, the median age was 33 years (IQR 26–43), and 42% (79/190) of the participants were HIV-infected.

### Resistance to first and second line drugs

No isolates (0/190, 0%, 95%CI 0.0–1.9%) were rifampicin-resistant using MTBDR*plus* and Xpert MTB/RIF, while one (0.5%, 95%CI 0.0–2.9%) was isoniazid-resistant using MTBDR*plus*. In a subset of 92 isolates, 71 (77%) had valid MTBDR*sl* assay results. None had detectable resistance to ethambutol or aminoglycosides/cyclic peptides, but 7 (9.8%, 95%CI 4.0–19.3%) were resistant to FQ on initial testing. However, the PCR products of the seven discordant isolates were sequenced and all had wild-type versions of FQ resistance genes. Both repeat MTBDR*sl* testing and MGIT culture testing confirmed them to be susceptible to FQ.

## Discussion

In a sample of 190 treatment-naïve patients with pulmonary TB in Southwestern Uganda, we found no rifampicin and minimal isoniazid (<1%) resistance using molecular-based resistance assays. In a sub-set of these specimens, no resistance to ethambutol or aminoglycosides was identified. FQ resistance using a molecular technique was detected but culture-based and sequencing assays did not confirm these results suggesting minimal resistance to FQs in this region as well. Our finding of low drug resistance rates to first line TB therapeutics is consistent with prior studies in Uganda. In a recent national drug survey, the MDR-TB prevalence was 1.4% among newly diagnosed patients [2]. In Southwestern Uganda, the prevalence was 1.6% in a similar population [7]. Similar rates were found in Rwanda (4.3%) [8], Kenya (3.2%) [9], and Tanzania (0.7%) [10]. These are the first reported data on first and second line drug susceptibility in Southwestern Uganda and support current MDR-TB treatment guidelines in Uganda, which recommend 6 months of daily kanamycin, levofloxacin, ethionamide, cycloserine and pyrazinamide followed by 18 months of daily levofloxacin, ethionamide, cycloserine and pyrazinamide as per WHO guidelines [6].

Despite frequent use of FQ in Uganda for respiratory infections per Ministry of Health guidelines [3], resistance among TB isolates appears to be rare. While we did detect resistance to FQ in 10% of isolates initially tested using the MTBDR*sl*, these results were not confirmed with either culture or sequencing based confirmatory methods. The laboratory was using this assay for the first time and found it very sensitive and prone to contamination. This could have contributed to the high false positive results.

Similarly, in a small sample of the Uganda national survey (n = 31), no FQ resistance was identified among MDR-TB patients [2]. The poor specificity of the MTBDR*sl* assay for FQ resistance has been reported previously. A study from the Democratic Republic of Congo reported a false positive rate of 57% for FQ resistance using this test [5]. Thus these results should promote caution in the use of the MTBDR*sl* assay for detection of FQs resistance and suggest the confirmation of resistance by the use of culture method where possible.

These results should be interpreted with the following limitations in mind. First, the study was limited to treatment-naïve individuals, so it does not represent resistance profiles from patients with treatment failure. Second, we primarily pursued molecular methods to estimate drug resistance. The sensitivity for drug resistance based on mutations in *kat*G (INH), *rpo*B (RIF) and MDR-TB has been estimated at ≥90%, ≥97%, and 99%, respectively [11]. Moreover, there is emerging data illustrating the complexity of TB drug resistance testing through pleomorphic drug effects, non-genetic resistance pathways, and discordance between genetic resistance and minimum inhibitory concentration thresholds [12]. Lastly, there was a high rate of PCR failure during testing with the MTBDR*sl* assay (23%), which might have contributed to under-estimation of resistance prevalence.

In conclusion, there was no evidence of first or second line drug resistance among treatment-naïve TB patients at Mbarara Regional Referral Hospital in Southwestern Uganda. These data support current national guidelines to include FQs, as empiric therapy for MDR-TB in the region. Future studies should investigate resistance patterns among previously treated patients and treatment failures, as well as evaluation of culture-based methods to corroborate our findings. Furthermore, molecular resistant testing should be routinely confirmed with culture based methods where possible.
